# Supplementary material for: Significant relaxation of SARS-CoV-2-targeted non-pharmaceutical interventions may result in profound mortality: A New York state modelling study
Source: PLoS One. 2020 Sep 24;15(9):e0239647. doi: 10.1371/journal.pone.0239647 (PMC7514073; doi:10.1371/journal.pone.0239647)
Supplement: S1 Appendix — (PDF) [file pone.0239647.s001.pdf]

SUPPLEMENTARY METHODS

System of Ordinary Differential Equations

$$\frac{dS}{dt} = -\chi(t)\rho\frac{\beta S}{N}(U + I) - \alpha(t)S + \zeta(t)P + \frac{\pi}{1-\tau}\left(R_U + R_Q + R_H\right)$$

$$\frac{dE}{dt} = \chi(t)\rho\frac{\beta S}{N}(U + I) - \gamma E$$

$$\frac{dI}{dt} = \gamma E - \nu I - \theta I - \delta I$$

$$\frac{dU}{dt} = \nu I - \phi U$$

$$\frac{dQ}{dt} = \theta I - \omega Q$$

$$\frac{dH}{dt} = \delta I - \lambda(t)H - \kappa(t)H$$

$$\frac{dR_U}{dt} = \phi U - \frac{\pi}{1-\tau}R_U$$

$$\frac{dR_Q}{dt} = \omega Q - \frac{\pi}{1-\tau}R_Q$$

$$\frac{dR_H}{dt} = \lambda(t)H - \frac{\pi}{1-\tau}R_H$$

$$\frac{dD}{dt} = \kappa(t)H$$

$$\frac{dP}{dt} = \alpha(t)S - \zeta(t)P$$

Parameter estimation

Model compartments Q, R_h, H, D were fit to extracted NYSDOH case data (confirmed, hospitalized total, hospitalized active, deaths) according to the following transformations: Q = (confirmed) – (hospitalized total); R_h = (hospitalized total) – (hospitalized active) – (deaths); H = (hospitalized active); D = (deaths). Parameters of the model were estimated with global optimization, using the particle swarm algorithm with post-hoc large-scale constrained non-linear minimization ("fmincon") implemented with an iterative sequential quadratic programming algorithm ("sqp") in Matlab (Tables S1-2) [1, 2]. Confidence intervals were estimated by bootstrapping the "fmincon" optimization algorithm with random lognormal gaussian noise applied to the SARS-CoV-2 sample data.

Sensitivity analysis and goodness of fit

The sensitivity of the model was estimated with two methods. First, bivariate sensitivity analysis was performed, where pairs of parameters were varied while other parameters were held at best-fit values. The model was simulated with each combination, and the total deaths of the simulation estimated by September 1st, 2020 were evaluated (Figure S2). Next, a global sensitivity analysis was performed to evaluate the first-order global sensitivity coefficients using the Sobol's method, with samples selected through 10000 iterations of a quasi-random Monte Carlo Simulation (Figure S3) [3]. Goodness of fit was evaluated by analyzing the residuals of the fit, which reveal an approximately normal distribution (Figure S4).

Reproductive number

The basic reproductive number (*R₀*) of the system was solved for using the next-generation matrix method (see, *Supplementary Methods*) [4, 5]:

$$R_0 = \frac{\chi\rho\beta}{\nu+\delta+\theta}(1+\frac{\nu}{\phi})$$

The instantaneous effective reproductive number (*R(t)*) was solved for with the following equation:

$$R(t) = \frac{R_o S}{N}$$

During the initial period of the outbreak of an infectious disease, there is relative lack of testing capacity compared to the prevalence of the pathogen. As testing capacity increases, one can confound this improved detection rate with rapid transmission of the pathogen (ascertainment bias). In order to avoid this, the peak reproductive number was estimated after the daily rate of change in the ratio of positive tests to confirmed tests was negative for 7 consecutive days. SARS-CoV-2 testing data was sourced from the NYSDOH [6].

Basic Reproductive Number Proof

To solve for the basic reproductive number, first the system was evaluated at the disease-free equilibrium:

$$\left(S_0, E_0, I_0, U_0, Q_0, H_0, R_{U_0}, R_{Q_0}, R_{H_0}, D_0, P_0\right) = (N, 0,0,0,0,0,0,0,0,0,0)$$

Next, a sub-model of the system was made where:

$$\frac{d\vec{x}}{dt} = F(\vec{x}) - V(\vec{x})$$

$$= F(\vec{x}) - (V^-(\vec{x}) - V^+(\vec{x}))$$

$$= \begin{bmatrix} \frac{\chi\rho\beta S(U+I)}{N} \\ 0 \\ 0 \\ 0 \\ 0 \end{bmatrix} - \begin{bmatrix} \gamma E \\ \nu I + \delta I + \theta I - \gamma E \\ \phi U - \nu I \\ \omega Q - \theta I \\ \lambda(t)H + \kappa(t)H - \delta I \end{bmatrix}$$

$$= \begin{bmatrix} \frac{\chi\rho\beta S(U+I)}{N} - \gamma E \\ \gamma E - \nu I - \delta I - \theta I \\ \nu I - \phi U \\ \theta I - \omega Q \\ \delta I - \lambda(t)H - \kappa(t)H \end{bmatrix}$$

Then, the Jacobian Matrix of the sub-model was evaluated at the disease-free equilibrium:

$$J\left(S_0, E_0, I_0, U_0, Q_0, H_0, R_{U_0}, R_{Q_0}, R_{H_0}, D_0, P_0\right) = \begin{bmatrix} -\gamma & \chi\rho\beta & \chi\rho\beta & 0 & 0 \\ \gamma & -\nu-\theta-\delta & 0 & 0 & 0 \\ 0 & \nu & -\phi & 0 & 0 \\ 0 & \theta & 0 & -\omega & 0 \\ 0 & \delta & 0 & 0 & -\lambda(t)-\kappa(t) \end{bmatrix}$$

Thus, the next generation matrix was:

$$FV^{-1} = \begin{bmatrix} \frac{\chi\rho\beta}{\nu+\delta+\theta}(1+\frac{\nu}{\phi}) & \frac{\chi\rho\beta}{\nu+\delta+\theta}(1+\frac{\nu}{\phi}) & \frac{\chi\rho\beta}{\phi} & 0 & 0 \\ 0 & 0 & 0 & 0 & 0 \\ 0 & 0 & 0 & 0 & 0 \\ 0 & 0 & 0 & 0 & 0 \\ 0 & 0 & 0 & 0 & 0 \end{bmatrix}$$

The basic reproductive number R_0 is the largest eigenvalue of the next generation matrix. Thus:

$$R_0 = \frac{\chi\rho\beta}{\nu+\delta+\theta}(1+\frac{\nu}{\phi})$$

Supplementary References

1. Coleman TF, Li Y. On the convergence of interior-reflective Newton methods for nonlinear minimization subject to bounds. Mathematical Programming. 1994;67(1-3):189-224. doi: 10.1007/bf01582221.

2. Kennedy J, Eberhart. R. Particle Swarm Optimization. Proceedings of the IEEE International Conference on Neural Networks. 1995:1942–5.

3. Sobol IM. On sensitivity estimation for nonlinear mathematical models. Matem Mod. 1990;2(1):112-8.

4. Diekmann O, Heesterbeek JA, Metz JA. On the definition and the computation of the basic reproduction ratio R0 in models for infectious diseases in heterogeneous populations. J Math Biol. 1990;28(4):365-82. Epub 1990/01/01. doi: 10.1007/BF00178324. PubMed PMID: 2117040.

5. Heffernan JM, Smith RJ, Wahl LM. Perspectives on the basic reproductive ratio. J R Soc Interface. 2005;2(4):281-93. Epub 2006/07/20. doi: 10.1098/rsif.2005.0042. PubMed PMID: 16849186; PubMed Central PMCID: PMCPMC1578275.

6. New York State Statewide COVID-19 Testing [Internet]. 2020. Available from: https://health.data.ny.gov/Health/New-York-State-Statewide-COVID-19-Testing/xdss-u53e.
