# Supplementary material for: Significant relaxation of SARS-CoV-2-targeted non-pharmaceutical interventions may result in profound mortality: A New York state modelling study
Source: PLoS One. 2020 Sep 24;15(9):e0239647. doi: 10.1371/journal.pone.0239647 (PMC7514073; doi:10.1371/journal.pone.0239647)
Supplement: S1 Table — (PDF) [file pone.0239647.s002.pdf]

S1 Table. Fixed Parameters

Parameter	Definition	Value	Details
ρ	Population Density (1000s people per mi^2)	2.726	[1]
γ^{-1}	Average incubation period	3	[2-4]
δ^{-1}	Average time to hospitalization	5	[5]
θ	Effective rate of non-hospitalized quarantine	0.57	Derived from source data [6, 7].
ν	Effective rate of undocumented infections	2.25	Undocumented rate of 0.75 constrained to g and d [8-10].
τ	Fraction non-immune	0.30 (varied in some simulations)	[11, 12]
π	Average duration of immunity	5 years (varied in some simulations)	[11, 12]

SUPPLEMENTARY REFERENCES

1. QuickFacts United States [Internet]. 2019 [cited 2019]. Available from: https://www.census.gov/quickfacts/.

2. Guan WJ, Ni ZY, Hu Y, Liang WH, Ou CQ, He JX, et al. Clinical Characteristics of Coronavirus Disease 2019 in China. N Engl J Med. 2020;382(18):1708-20. Epub 2020/02/29. doi: 10.1056/NEJMoa2002032. PubMed PMID: 32109013; PubMed Central PMCID: PMCPMC7092819.

3. Li Q, Guan X, Wu P, Wang X, Zhou L, Tong Y, et al. Early Transmission Dynamics in Wuhan, China, of Novel Coronavirus-Infected Pneumonia. N Engl J Med. 2020;382(13):1199-207. Epub 2020/01/30. doi: 10.1056/NEJMoa2001316. PubMed PMID: 31995857; PubMed Central PMCID: PMCPMC7121484.

4. Lauer SA, Grantz KH, Bi Q, Jones FK, Zheng Q, Meredith HR, et al. The Incubation Period of Coronavirus Disease 2019 (COVID-19) From Publicly Reported Confirmed Cases: Estimation and Application. Ann Intern Med. 2020. Epub 2020/03/10. doi: 10.7326/M20-0504. PubMed PMID: 32150748; PubMed Central PMCID: PMCPMC7081172.

5. Goyal P, Choi JJ, Pinheiro LC, Schenck EJ, Chen R, Jabri A, et al. Clinical Characteristics of Covid-19 in New York City. N Engl J Med. 2020. Epub 2020/04/18. doi: 10.1056/NEJMc2010419. PubMed PMID: 32302078; PubMed Central PMCID: PMCPMC7182018.

6. The Covid Tracking Project: New York State [Internet]. 2020 [cited 2020]. Available from: https://covidtracking.com/data/state/new-york.

7. New York State Statewide COVID-19 Testing [Internet]. 2020. Available from: https://health.data.ny.gov/Health/New-York-State-Statewide-COVID-19-Testing/xdss-u53e.

8. Bendavid E, Mulaney B, Sood N, Shah S, Ling E, Bromley-Dulfano R, et al. COVID-19 Antibody Seroprevalence in Santa Clara County, California. medRxiv. 2020:2020.04.14.20062463. doi: 10.1101/2020.04.14.20062463.

9. Sutton D, Fuchs K, D'Alton M, Goffman D. Universal Screening for SARS-CoV-2 in Women Admitted for Delivery. N Engl J Med. 2020. Epub 2020/04/14. doi: 10.1056/NEJMc2009316. PubMed PMID: 32283004; PubMed Central PMCID: PMCPMC7175422.

10. Cuomo A. Video, Audio, Photos & Rush Transcript: Amid Ongoing COVID-19 Pandemic, Governor Cuomo Announces State Health Department Will Partner with Attorney General James to Investigate Nursing Home Violations: Pressroom, NYS Governor’s Press Office; 2020 [cited 2020 4/23/2020]. Available from: https://www.governor.ny.gov/news/amid-ongoing-covid-19-pandemic-governor-cuomo-announces-state-health-department-will-partner.

11. Callow KA, Parry HF, Sergeant M, Tyrrell DA. The time course of the immune response to experimental coronavirus infection of man. Epidemiol Infect. 1990;105(2):435-46. Epub 1990/10/01. doi: 10.1017/s0950268800048019. PubMed PMID: 2170159; PubMed Central PMCID: PMCPMC2271881.

12. Chan KH, Chan JF, Tse H, Chen H, Lau CC, Cai JP, et al. Cross-reactive antibodies in convalescent SARS patients' sera against the emerging novel human coronavirus EMC (2012) by both immunofluorescent and neutralizing antibody tests. J Infect. 2013;67(2):130-40. Epub 2013/04/16. doi: 10.1016/j.jinf.2013.03.015. PubMed PMID: 23583636; PubMed Central PMCID: PMCPMC7112694.
