# Supplementary material for: Significant relaxation of SARS-CoV-2-targeted non-pharmaceutical interventions may result in profound mortality: A New York state modelling study
Source: PLoS One. 2020 Sep 24;15(9):e0239647. doi: 10.1371/journal.pone.0239647 (PMC7514073; doi:10.1371/journal.pone.0239647)
Supplement: S2 Table — (PDF) [file pone.0239647.s003.pdf]

S2 Table. Fit Parameters

Parameter	Definition	Value	95% Confidence Interval
α	Effective protection rate	0.105	(0.095, 0.112)
ζ	Effective protection leak rate	0.016	(0.012, 0.018)
β	Effective contact rate	0.281	(0.269, 0.285)
λ	Initial effective hospitalized recovery rate	0.174	(0.166, 0.200)
ε	Hospitalized recovery rate improvement rate constant	0.226	(0.191, 0.312)
σ	Efficacy of improved recovery rate	0.513	(0.405,0.575)
κ	Initial effective death rate	0.098	(0.092, 0.114)
ψ	Death rate improvement rate constant	0.404	(0.313, 0.438)
μ	Efficacy of reduced death rate	2.75	(2.465, 3.096)
