# Supplementary material for: Significant relaxation of SARS-CoV-2-targeted non-pharmaceutical interventions may result in profound mortality: A New York state modelling study
Source: PLoS One. 2020 Sep 24;15(9):e0239647. doi: 10.1371/journal.pone.0239647 (PMC7514073; doi:10.1371/journal.pone.0239647)
Supplement: S3 Table — (PDF) [file pone.0239647.s004.pdf]

S3 Table. Simulating the effect of undocumented infections on SARS-CoV-2 transmission, simulation results on September 1st, 2020, related to Figure 1.

Panel	Compartment	Value	95% Confidence Interval
A	Total Infections	1.53 x 10^6	(1.26 x 10^6, 1.79 x 10^6)
B	Undocumented Infections	1.15 x 10^6	(0.97 x 10^6, 1.34 x 10^6)
C	Confirmed Infections	3.82 x 10^5	(3.13 x 10^5, 4.50 x 10^5)
D	Active Confirmed Infections	500	(0, 2.9 x 10^3)
E	Total Hospitalized	1.00 x 10^5	(0.80 x 10^5, 1.20 x 10^5)
F	Total Deaths	2.85 x 10^4	(2.20 x 10^4, 3.76 x 10^4)
