# Supplementary material for: Significant relaxation of SARS-CoV-2-targeted non-pharmaceutical interventions may result in profound mortality: A New York state modelling study
Source: PLoS One. 2020 Sep 24;15(9):e0239647. doi: 10.1371/journal.pone.0239647 (PMC7514073; doi:10.1371/journal.pone.0239647)
Supplement: S4 Table — (PDF) [file pone.0239647.s005.pdf]

S4 Table. Reduction of NPIs by >50% may result increased transmission and significant mortality, simulation results on September 1st, 2020, related to Figure 2.

Panel	Compartment	NPI Reduction %	Value	95% Confidence Interval
A	Confirmed Active Infections	50	8.45 x 10^3	(0, 42.7 x 10^3)
		30	1.92 x 10^3	(0, 12.9 x 10^3)
		15	0.91 x 10^3	(0, 5.9 x 10^3)
B	Active Hospitalizations	50	1.38 x 10^3	(0, 8.75 x 10^3)
		30	0.27 x 10^3	(0, 2.53 x 10^3)
		15	0.11 x 10^3	(0, 1.17 x 10^3)
C	Cumulative Deaths	50	3.22 x 10^4	(2.32 x 10^4, 4.92 x 10^4)
		30	2.96 x 10^4	(2.26 x 10^4, 4.09 x 10^4)
		15	2.90 x 10^4	(2.23 x 10^4, 3.88 x 10^4)
G	Active Hospitalizations	75	3.33 x 10^4	(2.61 x 10^4, 3.53 x 10^4)
		25	0.02 x 10^4	(0, 0.19 x 10^4)
H	Cumulative Deaths	75	7.05 x 10^4	(3.12 x 10^4, 9.79 x 10^4)
		25	2.93 x 10^4	(2.25 x 10^4, 4.00 x 10^4)
