# Supplementary material for: Significant relaxation of SARS-CoV-2-targeted non-pharmaceutical interventions may result in profound mortality: A New York state modelling study
Source: PLoS One. 2020 Sep 24;15(9):e0239647. doi: 10.1371/journal.pone.0239647 (PMC7514073; doi:10.1371/journal.pone.0239647)
Supplement: S5 Table — (PDF) [file pone.0239647.s006.pdf]

S5 Table. Short-term effects of phased relaxed NPIs in NYS, simulation results on September 1st, 2020, related to Figure 2 and S8 Figure.

Panel	Compartment	NPI Reduction %	Value	95% Confidence Interval
A	Confirmed Active Infections	13→32→50	4.34 x 10^3	(0, 30.40 x 10^3)
		7→18→30	1.34 x 10^3	(0, 10.00 x 10^3)
		2→8→15	0.79 x 10^3	(0, 5.20 x 10^3)
B	Active Hospitalizations	13→32→50	0.71 x 10^3	(0, 6.07 x 10^3)
		7→18→30	0.20 x 10^3	(0, 1.95 x 10^3)
		2→8→15	0.10 x 10^3	(0, 1.02 x 10^3)
C	Cumulative Deaths	13→32→50	3.00 x 10^4	(2.27 x 10^4, 4.30 x 10^4)
		7→18→30	2.91 x 10^4	(2.24 x 10^4, 3.93 x 10^4)
		2→8→15	2.88 x 10^4	(2.22 x 10^4, 3.83 x 10^4)
G	Active Hospitalizations	22→48→75	1.14 x 10^4	(0, 3.46 x 10^4)
		5→15→25	0.02 x 10^4	(0, 0.15 x 10^4)
H	Cumulative Deaths	22→48→75	3.82 x 10^4	(2.38 x 10^4, 6.67 x 10^4)
		5→15→25	2.89 x 10^4	(2.23 x 10^4, 3.89 x 10^4)
