# Supplementary figures and images for: Significant relaxation of SARS-CoV-2-targeted non-pharmaceutical interventions may result in profound mortality: A New York state modelling study
Source: PLoS One. 2020 Sep 24;15(9):e0239647. doi: 10.1371/journal.pone.0239647 (PMC7514073; doi:10.1371/journal.pone.0239647)

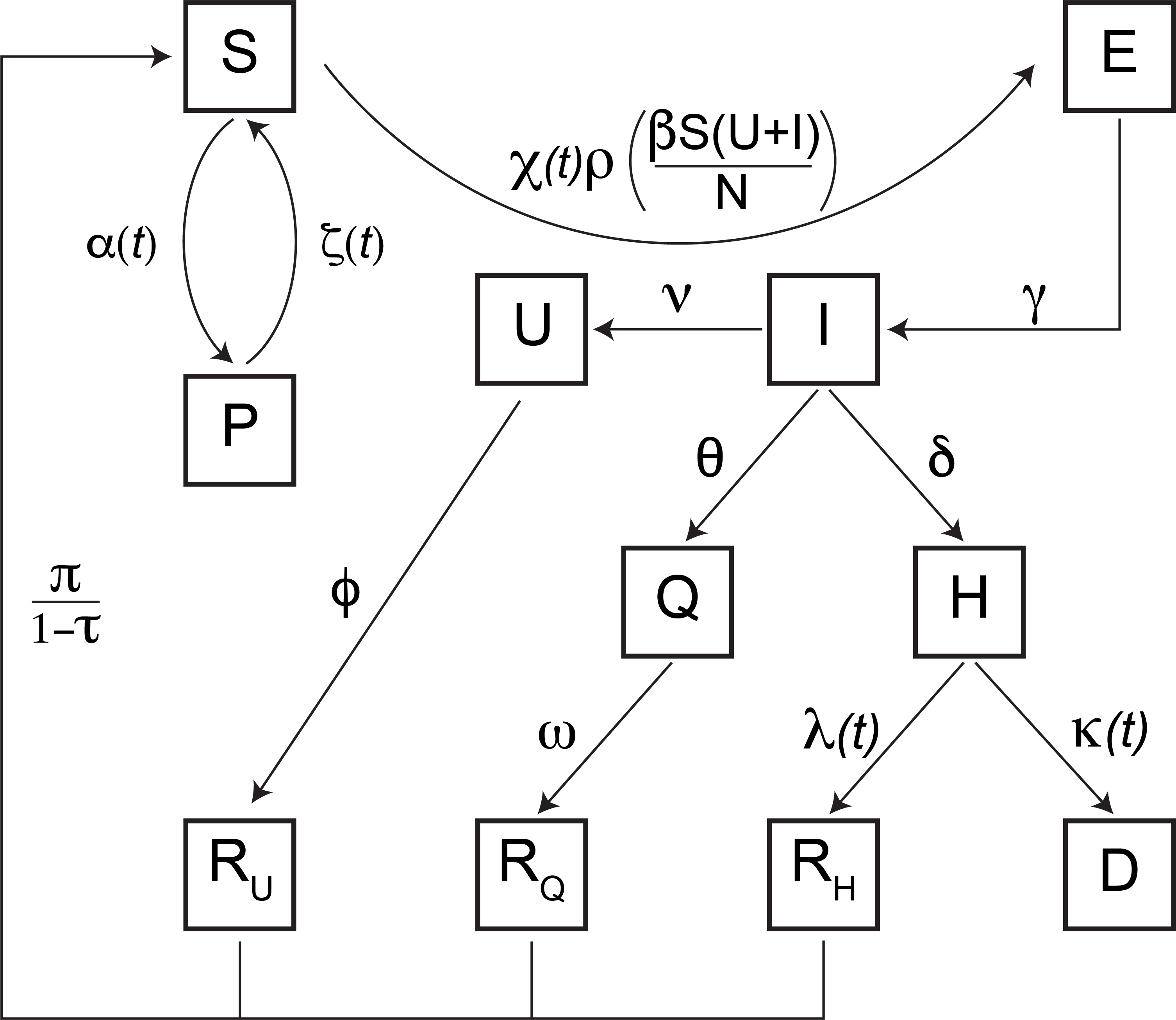

Supplement: S1 Fig — S (susceptible), E (exposed individuals), I (infected), U (undocumented), Q (quarantined), H (hospitalized), RU (recovered undocumented), RQ (recovered quarantined), RH (recovered hospitalized), D (dead), and P (protected). (PNG) [file pone.0239647.s007.png]

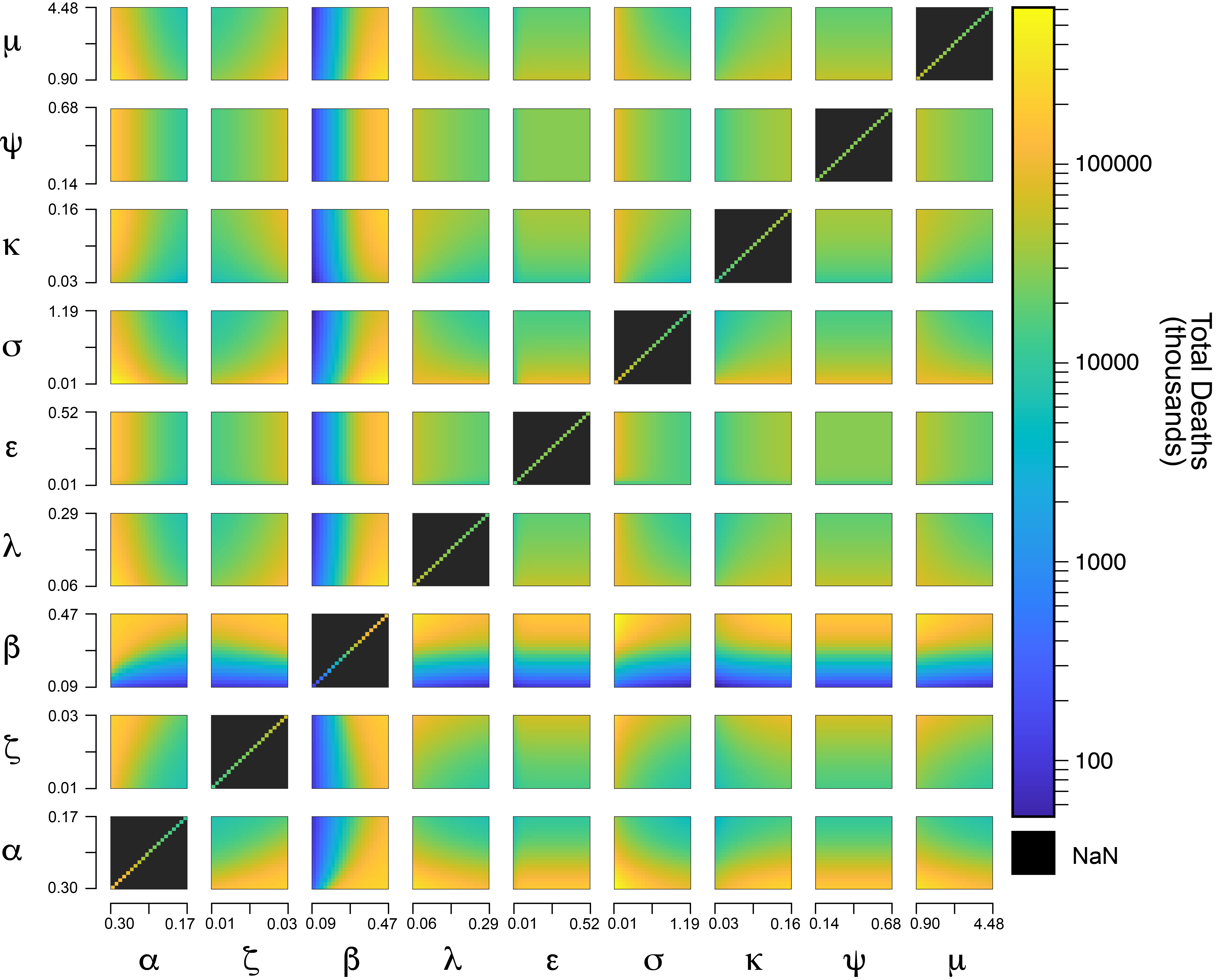

Supplement: S2 Fig — (PNG) [file pone.0239647.s008.png]

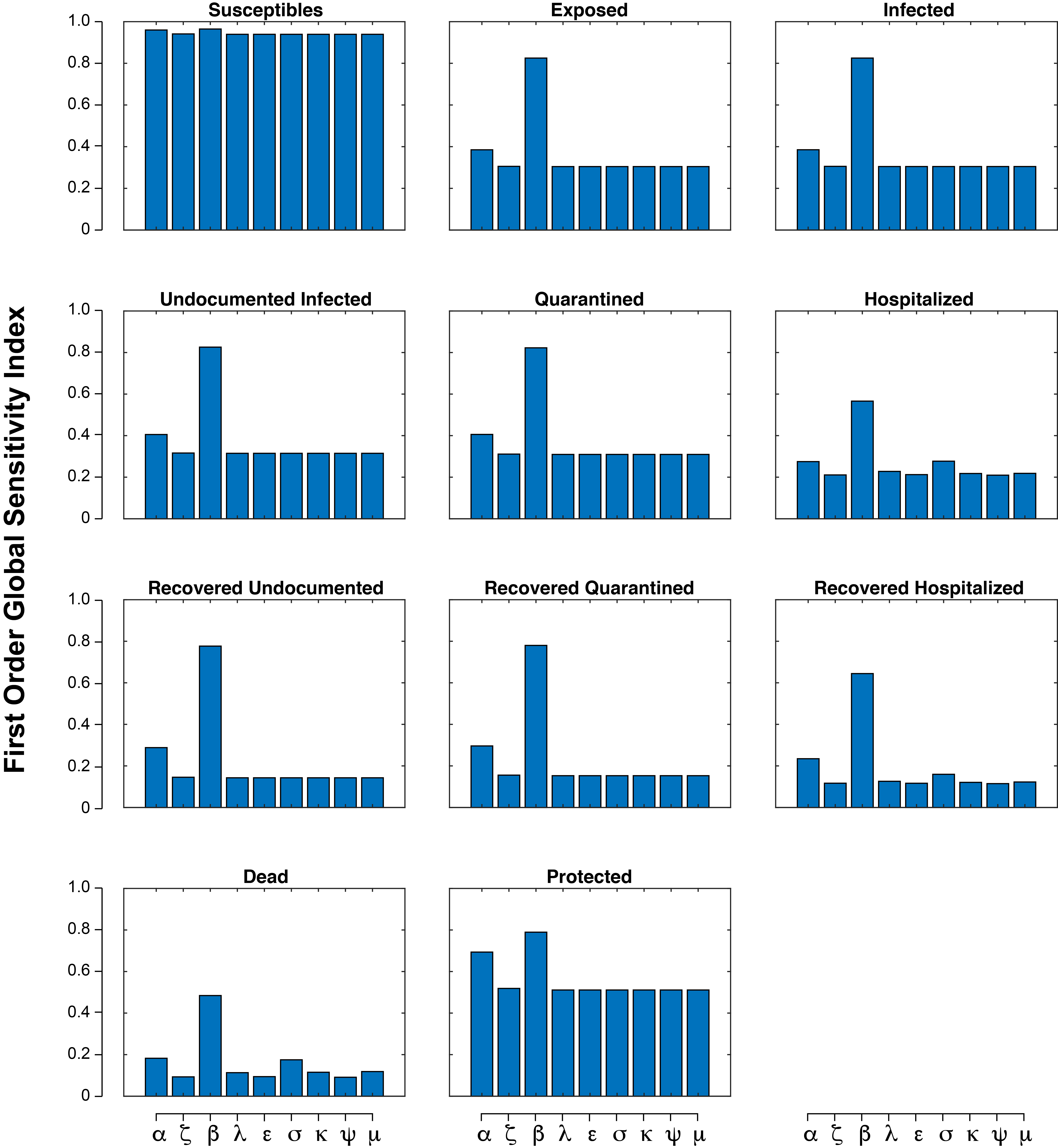

Supplement: S3 Fig — (PNG) [file pone.0239647.s009.png]

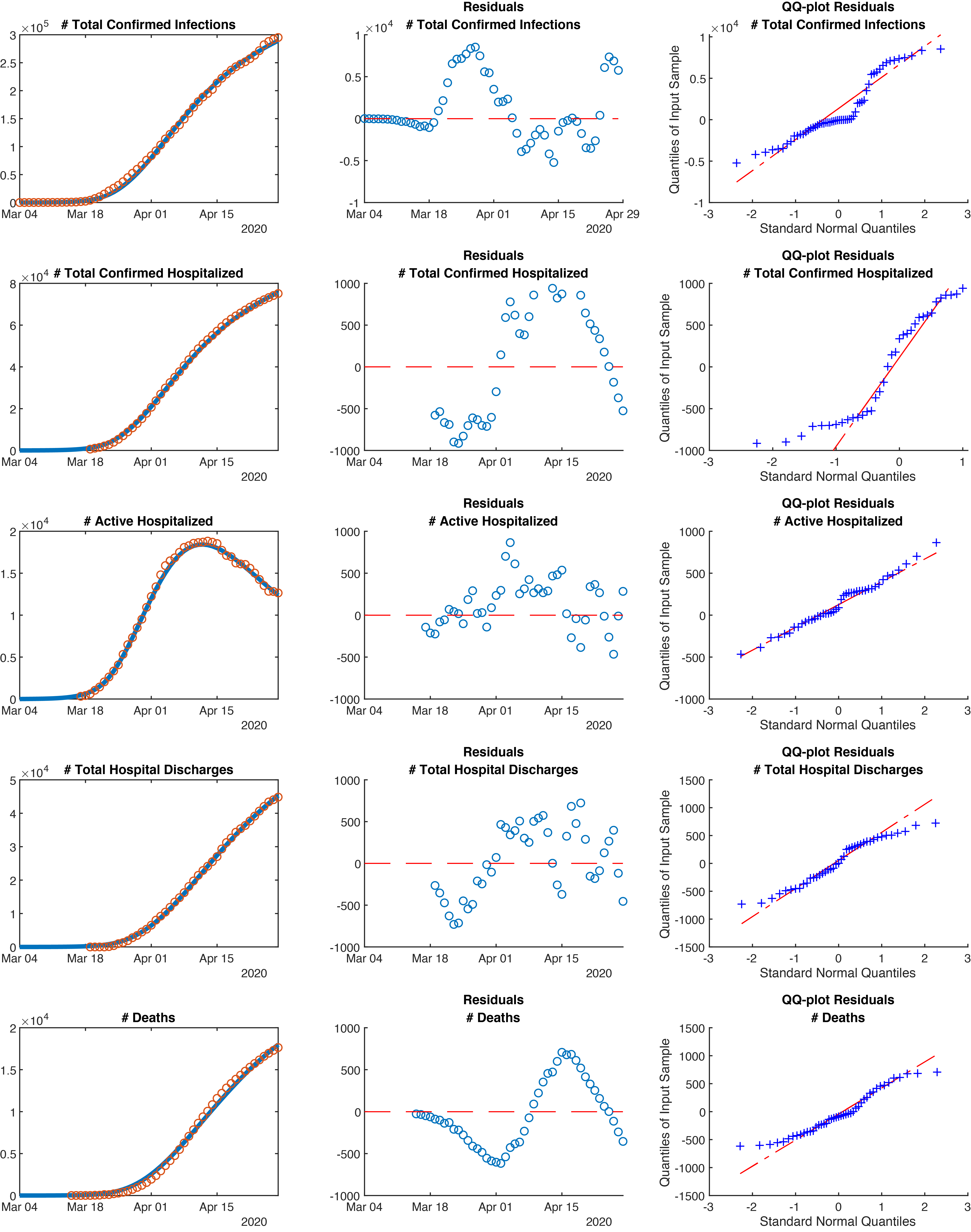

Supplement: S4 Fig — (PNG) [file pone.0239647.s010.png]

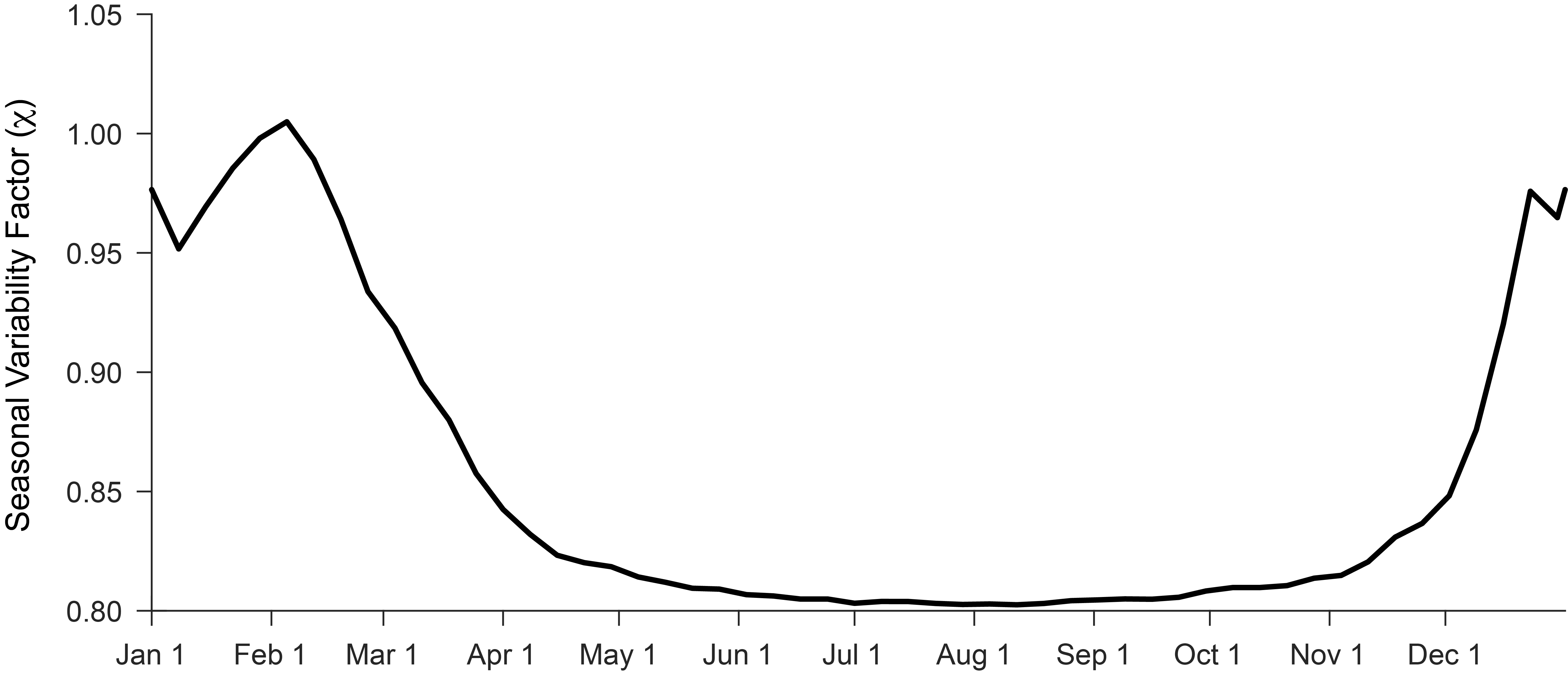

Supplement: S5 Fig — (PNG) [file pone.0239647.s011.png]

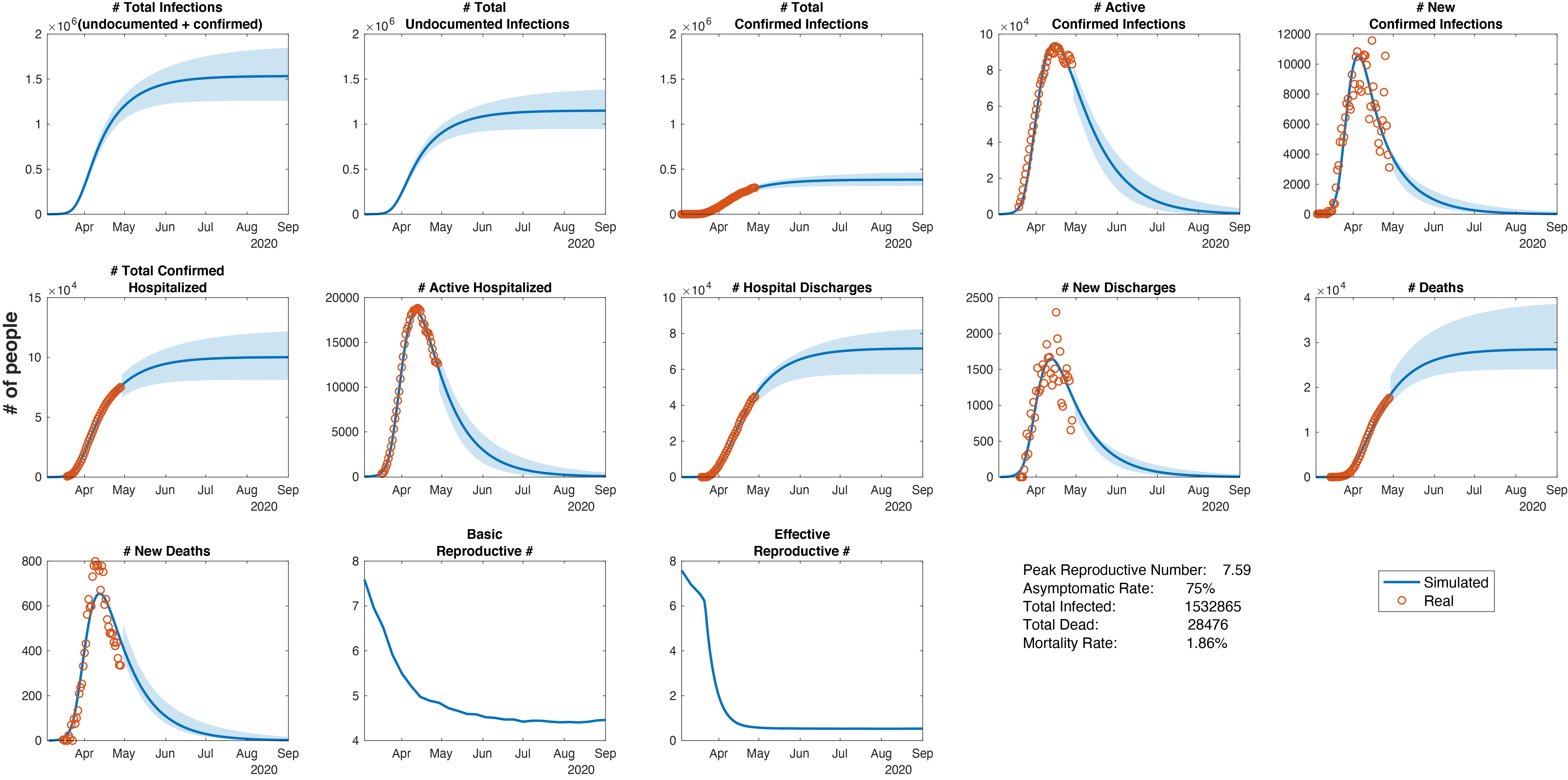

Supplement: S6 Fig — (PNG) [file pone.0239647.s012.png]

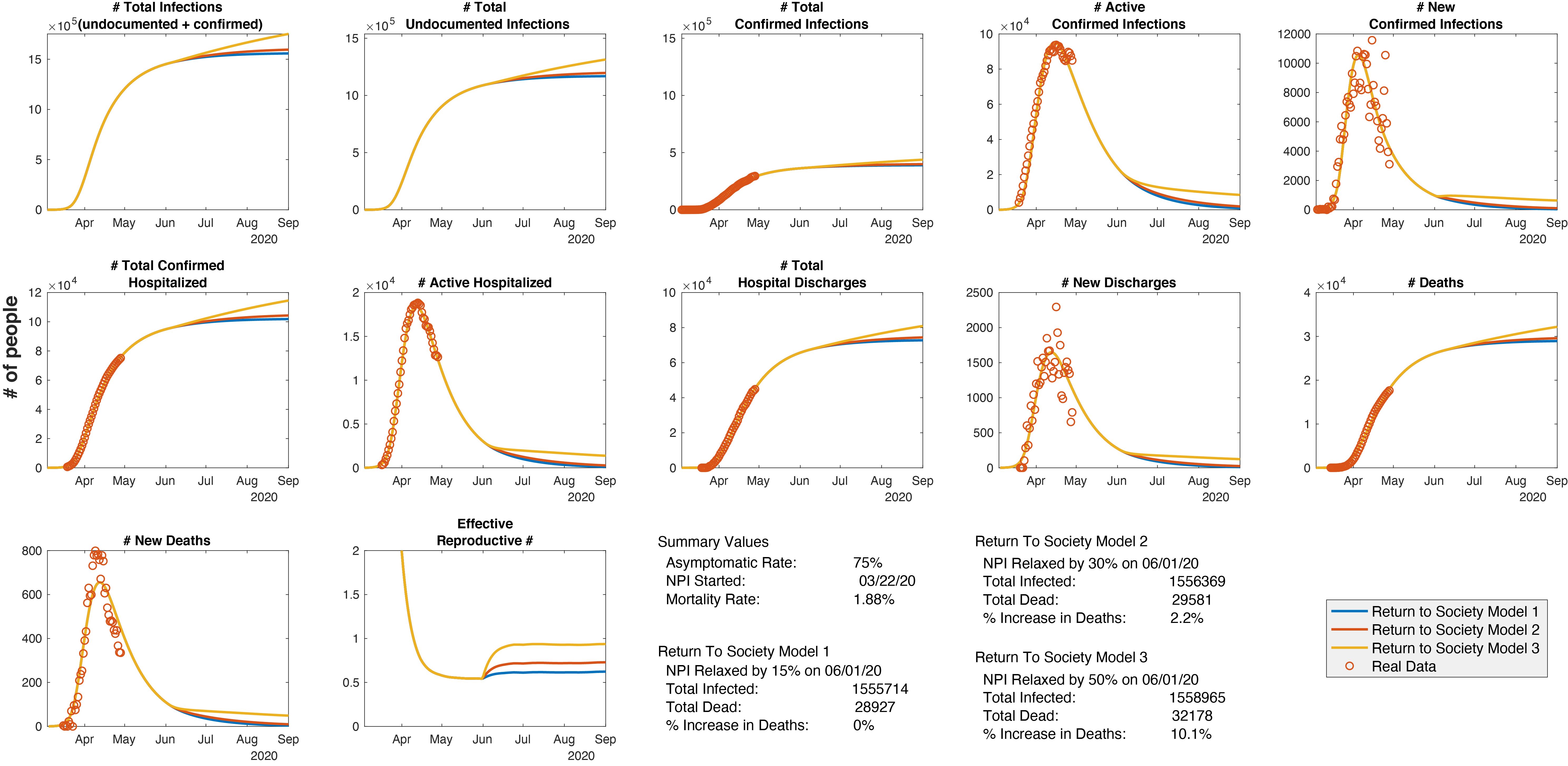

Supplement: S7 Fig — (PNG) [file pone.0239647.s013.png]

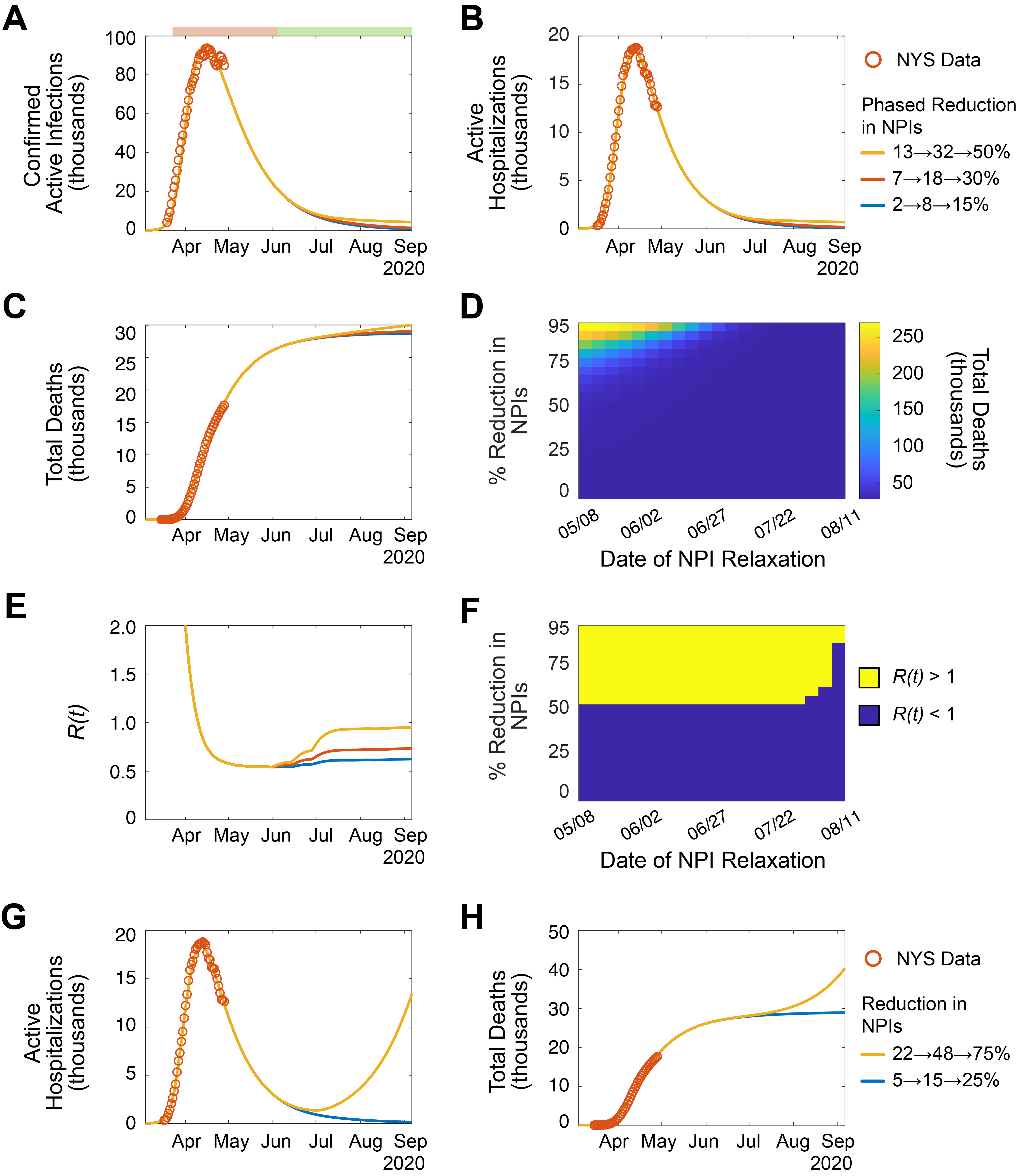

Supplement: S8 Fig — (A-F). Simulation of SARS-CoV-2 transmission dynamics in the presence of NPIs through September 1, 2020. Periods of NPIs signified as in (A) top: pink, increased NPIs; green, relaxed NPIs. Orange circles, NYS SARS-CoV-2 data. Lines, simulated projection of reduced NPIs starting June 1, 2020. A. Active confirmed infections. B. Active hospitalizations. C. Cumulative deaths. D. Heatmap displaying the effect of NPI magnitude and date of reduction on the number of cumulative deaths. E. R(t). F. Categorical heatmap displaying the effect of NPI magnitude and date of reduction on R(t) > 1 (yellow, R(t)> 1; blue R(t)< 1). (G-H). Simulation of extreme reduction of NPIs on June 1, 2020. G. Active hospitalizations. H. Cumulative deaths. (PNG) [file pone.0239647.s014.png]

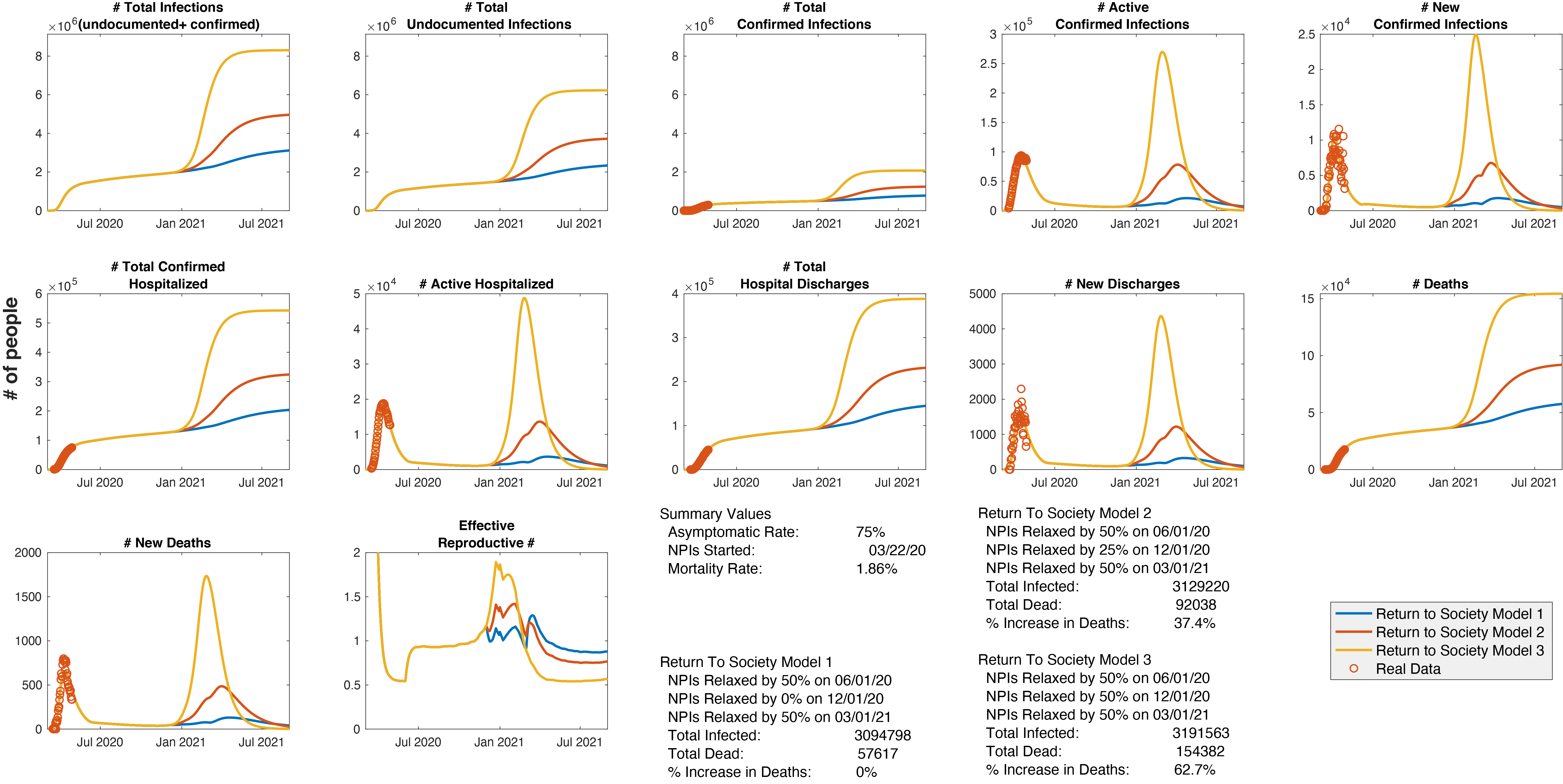

Supplement: S9 Fig — (PNG) [file pone.0239647.s015.png]

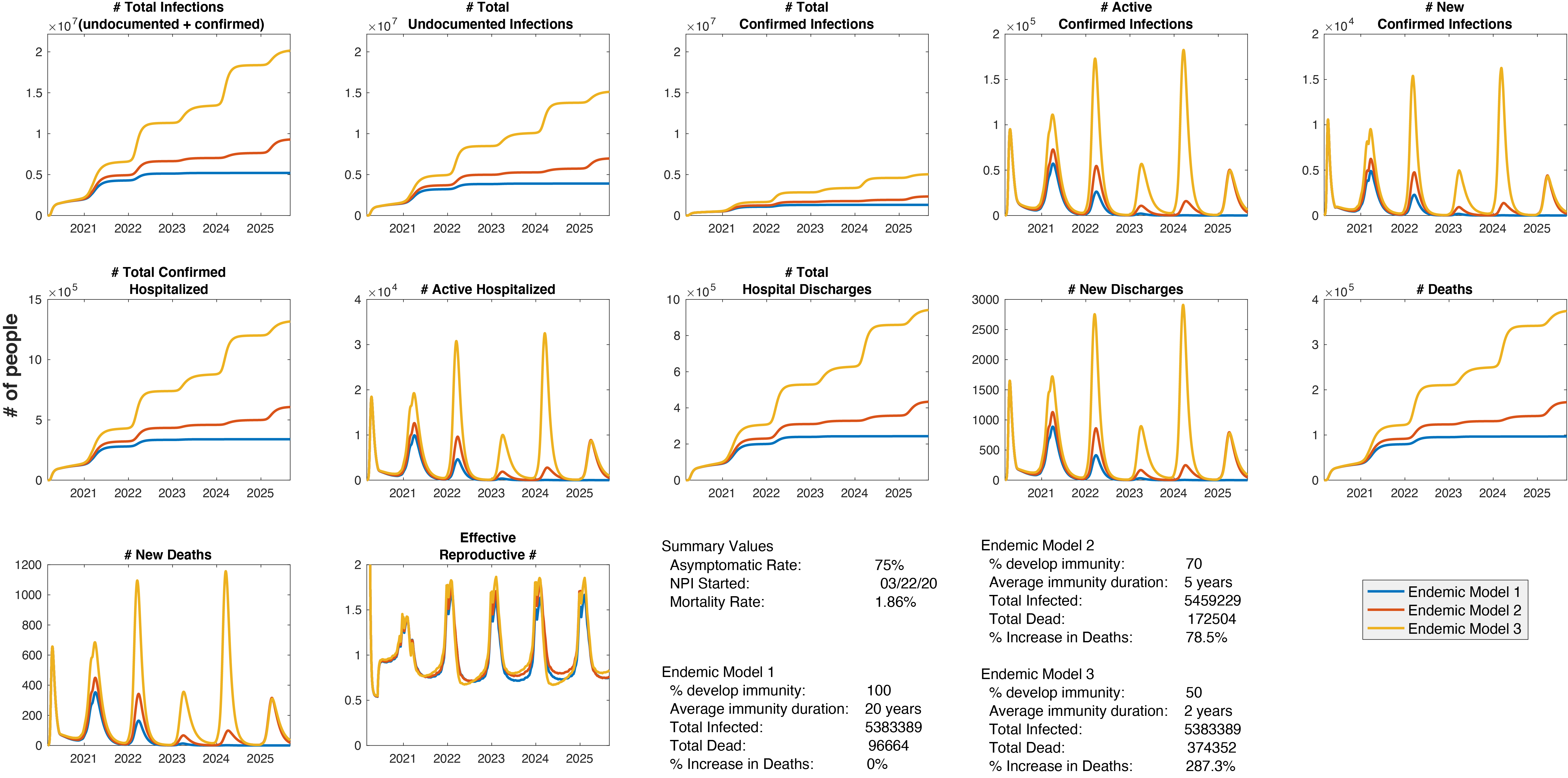

Supplement: S10 Fig — (PNG) [file pone.0239647.s016.png]
